# Accuracy of Practitioner Estimates of Probability of Diagnosis Before and After Testing

**DOI:** 10.1001/jamainternmed.2021.0269

**Published:** 2021-04-05

**Authors:** Daniel J. Morgan, Lisa Pineles, Jill Owczarzak, Larry Magder, Laura Scherer, Jessica P. Brown, Chris Pfeiffer, Chris Terndrup, Luci Leykum, David Feldstein, Andrew Foy, Deborah Stevens, Christina Koch, Max Masnick, Scott Weisenberg, Deborah Korenstein

**Affiliations:** 1 Department of Epidemiology and Public Health, University of Maryland School of Medicine, Baltimore; 2 Veterans Affairs (VA) Maryland Healthcare System, Baltimore; 3 Department of Health, Behavior, and Society, Johns Hopkins Bloomberg School of Public Health, Baltimore, Maryland; 4 Adult and Child Consortium of Health Outcomes Research and Delivery Science, University of Colorado School of Medicine, Aurora; 5 Division of Cardiology, University of Colorado School of Medicine, Aurora; 6 Center of Innovation for Veteran-Centered and Value-Driven Care, VA Denver, Denver, Colorado; 7 Division of General Internal Medicine & Geriatrics, Department of Medicine, Oregon Health & Science University, Portland; 8 Department of Medicine, Dell Medical School, the University of Texas at Austin, Austin; 9 Department of Medicine, South Texas Veterans Health Care System, San Antonio; 10 Department of Medicine, University of Wisconsin School of Medicine and Public Health, Madison; 11 Department of Medicine, Penn State College of Medicine, Hershey, Pennsylvania; 12 Department of Public Health Sciences, Penn State College of Medicine, Hershey, Pennsylvania; 13 Department of Medicine, University of Maryland School of Medicine, Baltimore; 14 Department of Informatics, Genomic Medicine Institute, Geisinger, Danville, Pennsylvania; 15 Division of Infectious Diseases, New York University Grossman School of Medicine, New York; 16 Division of General Internal Medicine, Memorial Sloan Kettering Cancer Center, New York, New York

## Abstract

**Importance:**

Accurate diagnosis is essential to proper patient care.

**Objective:**

To explore practitioner understanding of diagnostic reasoning.

**Design, Setting, and Participants:**

In this survey study, 723 practitioners at outpatient clinics in 8 US states were asked to estimate the probability of disease for 4 scenarios common in primary care (pneumonia, cardiac ischemia, breast cancer screening, and urinary tract infection) and the association of positive and negative test results with disease probability from June 1, 2018, to November 26, 2019. Of these practitioners, 585 responded to the survey, and 553 answered all of the questions. An expert panel developed the survey and determined correct responses based on literature review.

**Results:**

A total of 553 (290 resident physicians, 202 attending physicians, and 61 nurse practitioners and physician assistants) of 723 practitioners (76.5%) fully completed the survey (median age, 32 years; interquartile range, 29-44 years; 293 female [53.0%]; 296 [53.5%] White). Pretest probability was overestimated in all scenarios. Probabilities of disease after positive results were overestimated as follows: pneumonia after positive radiology results, 95% (evidence range, 46%-65%; comparison *P* < .001); breast cancer after positive mammography results, 50% (evidence range, 3%-9%; *P* < .001); cardiac ischemia after positive stress test result, 70% (evidence range, 2%-11%; *P* < .001); and urinary tract infection after positive urine culture result, 80% (evidence range, 0%-8.3%; *P* < .001). Overestimates of probability of disease with negative results were also observed as follows: pneumonia after negative radiography results, 50% (evidence range, 10%-19%; *P* < .001); breast cancer after negative mammography results, 5% (evidence range, <0.05%; *P* < .001); cardiac ischemia after negative stress test result, 5% (evidence range, 0.43%-2.5%; *P* < .001); and urinary tract infection after negative urine culture result, 5% (evidence range, 0%-0.11%; *P* < .001). Probability adjustments in response to test results varied from accurate to overestimates of risk by type of test (imputed median positive and negative likelihood ratios [LRs] for practitioners for chest radiography for pneumonia: positive LR, 4.8; evidence, 2.6; negative LR, 0.3; evidence, 0.3; mammography for breast cancer: positive LR, 44.3; evidence range, 13.0-33.0; negative LR, 1.0; evidence range, 0.05-0.24; exercise stress test for cardiac ischemia: positive LR, 21.0; evidence range, 2.0-2.7; negative LR, 0.6; evidence range, 0.5-0.6; urine culture for urinary tract infection: positive LR, 9.0; evidence, 9.0; negative LR, 0.1; evidence, 0.1).

**Conclusions and Relevance:**

This survey study suggests that for common diseases and tests, practitioners overestimate the probability of disease before and after testing. Pretest probability was overestimated in all scenarios, whereas adjustment in probability after a positive or negative result varied by test. Widespread overestimates of the probability of disease likely contribute to overdiagnosis and overuse.

## Key Points

**Question:**

Do practitioners understand the probability of common clinical diagnoses?

**Findings:**

In this survey study of 553 practitioners performing primary care, respondents overestimated the probability of diagnosis before and after testing. This posttest overestimation was associated with consistent overestimates of pretest probability and overestimates of disease after specific diagnostic test results.

**Meaning:**

These findings suggest that many practitioners are unaccustomed to using probability in diagnosis and clinical practice. Widespread overestimates of the probability of disease likely contribute to overdiagnosis and overuse.

## Teaser

This survey study of physicians, nurse practitioners, and physician assistants explores practitioner understanding of diagnostic reasoning.

## Introduction

Diagnosis of disease is complex and taught using estimated probabilities based on the patient’s history, physical examination findings, and diagnostic test results.^1,2,3^ Correct ordering and interpretation of tests are increasingly important given the increase in the number and complexity of tests, with more than 14 billion tests performed yearly in the US alone.^4^ Although practitioners are taught to estimate pretest probability and to apply the sensitivity and specificity of a test to interpret a positive or negative result, data suggest that historically most practitioners perform poorly on assessments of these skills and do not use these approaches in day-to-day practice.^5,6,7,8,9,10,11^

Test ordering and interpretation are taught briefly in medical schools,^12^ with curricular evaluation often limited to self-assessment of skills.^13^ The impact of such education on clinical practice is unclear. Estimating the probability of disease and deciding to test may be influenced by training, experience, and personality.^8,14^ Medical decisions, like other human decisions, may not be rational and are prone to errors associated with poor knowledge of the base rate of disease or other errors associated with probability.^14^ Test performance and interpretation have increasingly become a point of discussion in medicine and for the general public during the COVID-19 pandemic.^15^ Erroneous estimates of disease probability likely impact practitioner treatment decisions.^3,16^ Lack of accurate diagnostic reasoning may lead to overdiagnosis and overtreatment.^17^

Few studies have systematically examined how practitioners interpret diagnostic test results within the context of actual clinical scenarios. We performed a multicenter survey of practitioners in primary care practice to explore practitioner understanding of the probability of disease before and after test results for common clinical scenarios.

## Methods

### Survey

We developed a survey to assess practitioner test understanding and the process of making a diagnosis using probability as well as actions taken by practitioners in similar scenarios in their practice. The survey also included items regarding basic demographic characteristics, educational background, and practice setting. Institutional review board approval was obtained at each of the 3 coordinating sites (Baltimore, Maryland; San Antonio, Texas; and Portland, Oregon). Verbal informed consent with a waiver of documentation was approved at all sites. The study followed the American Association for Public Opinion Research (AAPOR) reporting guideline.

A draft survey was developed by primary investigators (D.J.M., L.L., D.K., D.F., L.S., J.P.B., A.F., S.W., C.P., J.O., and L.P.) based in part on previous surveys of risk understanding.^5,8,9,10,11,18^ This survey was reviewed by an expert panel of practitioners with different areas of expertise, practicing in community and academic settings (D.J.M., L.L., D.F., A.F., S.W., and D.K.), a qualitative research expert (J.O.), an epidemiologist (J.P.B.) and a psychologist (L.S.) with expertise in survey design, and a senior biostatistician (L.M.). The survey was further revised by the expert panel during an in-person meeting and 2 conference calls. A pilot test of the survey was conducted with 10 practitioners for comprehension and interpretation of questions, and minor language adjustments were made.

### Practitioner Risk Understanding

The survey assessed risk understanding for common testing clinical decisions encountered by primary care practitioners in routine scenarios similar to previous small surveys.^8,9,10,11,18^ Individual testing questions pertained to mammograms for breast cancer, stress testing for cardiac ischemia, chest radiography for pneumonia, and urine cultures for urinary tract infection (UTI) (eAppendix 1 in the Supplement).

Practitioners were presented with a clinical scenario and asked to estimate pretest probability of disease and posttest probabilities after both positive and negative test results. Each scenario was created for a general situation but included essential details to calculate true risk for patients (eg, age and absence of any risk factors for breast cancer in mammogram screening questions). The primary outcome of testing questions was to accurately identify the probability that a patient had disease after positive or negative results. Questions were designed to assess whether errors in test interpretation associated with poor pretest estimates or inaccurate updating of probability after testing. Additional questions provided sensitivity and specificity of a theoretical test and asked participants to calculate positive and negative predictive value at particular levels of disease prevalence.

To assess the accuracy of participant responses, we used a hierarchical method to identify the scientific evidence for pretest probability, sensitivity, and specificity from the literature, which was completed after survey finalization. We first reviewed high-quality recent systematic reviews and meta-analyses. If only older systematic reviews and meta-analyses were available, with newer high-impact studies after publication, we considered data from both (attempting to understand the most accurate numbers for current technology and practice). If no systematic reviews or meta-analyses were available, we used data from studies commonly cited in recent guidelines, creating weighted means by consensus. The expert panel of physicians overseeing the study was presented with the best evidence identified, had a comment and question period, and determined consensus evidence-based answers presented in the Results section (eAppendix 2 in the Supplement).

### Enrollment Procedure

People in leadership positions for group practices or residency programs were contacted and informed of the study. Investigators sought permission to give a short presentation or email introduction that described the study during a group practice meeting. Individual practitioners were then approached by a coordinator and/or physician investigator to request participation. The survey was offered to 723 primary care physicians, nurse practitioners, and physician assistants practicing in Delaware, Maryland, Oregon, Pennsylvania, Texas, Virginia, Washington, and the District of Columbia (Table 1). The survey was administered in paper format. The coordinator generally remained at the clinic, office, or meeting location until the practitioner had completed the survey. If practitioners requested to complete the survey at a later date, they were provided with an addressed, stamped envelope and could return the survey by mail, email, or clinic drop-off. Respondents were provided with a US $50 gift card for completion, if permitted by their employer.

**Table 1.** Survey Responses

Variable	No. (%) of practitioners			
	Maryland and Middle Atlantic states	Oregon and Washington	Texas	All sites
Invited to participate	390	150	183	723
No response	41 (11)	0	16 (9)	57 (8)
Refusals^a^	10 (3)	3 (2)	3 (2)	16 (2)
Not interested	2	0	2	4
Too busy or bad timing	6	2	2	10
Too difficult	2	1	0	3
Other	3	0	0	3
Agreed to participate (of all invited)	339 (87)	147 (98)	164 (90)	650 (90)
Agreed but did not complete survey	27 (7)	23 (15)	15 (8)	65
Total surveys received	312 (80)	124 (83)	149 (81)	585 (81)
Failed to complete ≥1 questions required for final analysis	7 (2)	12 (8)	13 (7)	32 (4)

^a^ May list more than 1 reason for refusing to complete the survey.

Practitioners who initially agreed to participate but did not return the survey within 2 weeks were contacted by study staff via email and/or in person up to 5 times during 3 months. Practitioners who did not complete the survey after these subsequent contacts were considered nonparticipants. Practitioners who declined to participate at initial enrollment or after reminders were asked to provide a reason for not participating from a standardized list to assess for selection bias. Of the contacted practitioners, 585 responded to the survey, and 553 answered all the questions.

### Imputed Likelihood Ratios

To understand the adjustment in probability of disease after a positive or negative test result, we calculated an imputed likelihood ratio.^19^ By comparing estimated probability of disease before and after testing, we could impute the likelihood ratio that was consciously or unconsciously applied to modify probabilities. The imputed likelihood ratio was calculated by dividing posttest odds by pretest odds, where odds were calculated as probability divided by 1 minus probability.^19^ Responses of 0% or 100% were modified to 0.1% and 99.9% to allow for calculation of a likelihood ratio. Likelihood ratios were estimated from the literature as described above by the expert panel of physicians (eAppendix 2 in the Supplement).

### Statistical Analysis

Survey responses were entered into a REDCap (Research Electronic Data Capture) database with double data entry. A sample size of 500 was planned based on desire for generalizable results across enrollment sites. The target sample was surpassed while we collected outstanding surveys. Data were analyzed with R software (R Foundation for Statistical Computing) for creation of density plots. SAS statistical software, version 9.4 (SAS Institute Inc) was used for calculation of descriptive statistics and all other statistical analyses. Comparison of those who completed all key survey questions with those who did not was performed with the χ^2^ test. To assess the statistical significance of differences between respondent estimates of diagnostic probabilities and the probabilities determined from scientific evidence, we used Wilcoxon signed-rank tests. To display the range of results for estimates of probability, we used density plots. These were created using R software (GGPlot2). A 2-sided *P* < .05 was considered to be statistically significant.

## Results

### Participant Demographics

A total of 553 of 723 practitioners (76.5%) fully completed the survey (median age, 32 years; interquartile range, 29-44 years; 293 female [53.0%]; 296 [53.5%] White) from June 1, 2018, to November 26, 2019 (Table 2). A total of 492 of the 553 respondents (89.0%) had MD or DO degrees, and 290 (52.4% were in residency). The survey required a median of 20 minutes to complete (interquartile range [IQR], 15-25 minutes).

**Table 2.** Variables Associated With Practice Among Enrolled Practitioners

Variable	No. (%) of respondents
Practitioner type	
MD or equivalent	492 (89.0)
NP	48 (8.7)
PA	13 (2.4)
Race/ethnicity	
White	296 (53.5)
Black	37 (6.7)
Asian	142 (25.7)
Hispanic/Latino	45 (8.1)
>1 Race/ethnicity	19 (3.4)
Other or missing	14 (2.5)
Female sex	293 (53.0)
Age, median (IQR), y	32 (29.0-44.0)
Medical, nursing, or PA school	
International	113 (20.4)
Osteopathy school	21 (3.8)
Current resident	290 (52.4)
Type of residency	
Internal medicine	336 (60.8)
Family medicine	142 (25.7)
Other or NA	75 (13.6)
Type of practice (n = 633; may be >1 type)	
Academic	343 (54.2)
Rural	7 (1.1)
Suburban	60 (9.5)
Urban	83 (13.1)
VA	141 (22.3)
Ever sued for malpractice	31 (5.6)
Other graduate degree	115 (20.8)
Time in practice, median (IQR), y	3 (1.0-10.0)
Resident	1.5 (1.0-3.0)
Nonresident	11 (5.0-22.0)

Abbreviation: IQR, interquartile range; MD, physician; NA, not applicable; NP, nurse practitioner; PA, physician assistant; VA, Veterans Affairs.

We compared the 32 respondents who did not complete all necessary questions with the final cohort of 553 practitioners with complete responses. We found that those not completing the survey were more likely to be female (26 [81.3%] noncompleters vs 293 [53.0%] final cohort, *P* < .001), to have been in practice more than 10 years (15 [46.9%] noncompleters vs 145 [26.2%] final cohort, *P* = .01), to be nonresidents (27 [84.4%] noncompleters vs 263 [47.6%] final cohort, *P* < .001), or to be nurse practitioners or physicians assistants (13 [40.6%] noncompleters vs 61 [11.0%] final cohort, *P* < .001).

### Estimates of Disease Probability

Estimates of probability of disease were consistently higher than scientific evidence (Figure). We also broke down answers by type of practitioner (resident physician, attending physician, and nurse practitioner or physician assistant) (Table 3). All types of practitioners overestimated probability of disease before and after testing.

**Figure.**
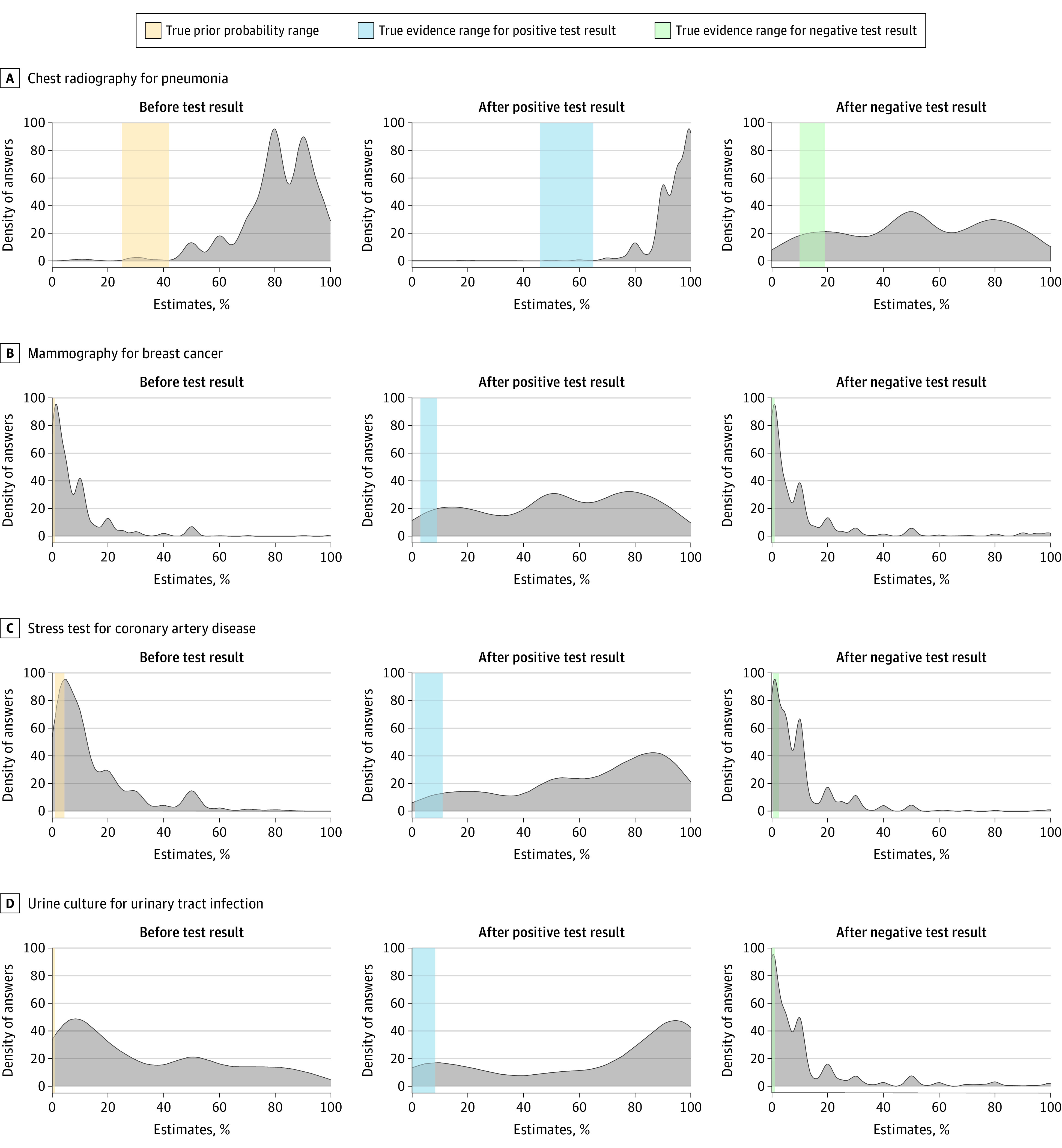
Distribution of Practitioner Assessments of Probability of Disease Before Testing and After Positive or Negative Test Results for 4 Testing Questions Representing Scenarios Commonly Encountered in Primary Care A, Scenario: a previously healthy 35-year-old woman who smokes tobacco presents with 5 days of fatigue, productive cough, worsening shortness of breath, temperatures to 38.9°C, and decreased breath sounds in the lower right field. She has a heart rate of 105 beats/min, but vital signs are otherwise normal. B, Scenario: a 45-year-old woman comes in for an annual visit. She has no specific risk factors or symptoms for breast cancer. C, Scenario: a 43-year-old premenopausal woman presents with atypical chest pain and normal ECG results. She has no risk factors and has normal vital signs and examination findings. D, Scenario: a 65-year-old man is seen for osteoarthritis. He has noted foul-smelling urine and no pain or difficulty with urination. A urine dipstick shows trace blood. ECG indicates electrocardiography.

**Table 3.** Estimates of Probability of Disease Before Testing and After Positive or Negative Test Results for 5 Testing Questions

Clinical scenario	Scientific evidence range, %	Median (IQR)		
		Resident physician estimate, % (n = 290)	Attending physician estimate, % (n = 202)	Nurse practitioner or physician assistant estimate, % (n = 61)
Pneumonia				
Pretest probability	25-42	80 (75-90)	85 (80-90)	80 (70-90)
After positive test result	46-65	95 (90-99)	95 (95-100)	95 (90-100)
After negative test result	10-19	60 (40-80)	50 (20-80)	50 (20-50)
Breast cancer				
Pretest probability	0.2-0.3	5 (1-10)	2 (1-10)	10 (5-20)
After positive test result	3-9	60 (35-75)	50 (20-80)	60 (50-80)
After negative test result	<0.05	5 (1-10)	1 (1-10)	10 (2-20)
Cardiac ischemia				
Pretest probability	1-4.4	10 (5-20)	5 (3-10)	15 (6.25-30)
After positive test result	2-11	75 (50-90)	60 (25-80)	90 (60-95)
After negative test result	0.43-2.5	5 (1-10)	5 (1-10)	10 (5-20)
Urinary tract infection				
Pretest probability	0-1	25 (10-60)	20 (5-50)	30 (10-50)
After positive test result	0-8.3	77.5 (25-95)	90 (40-98)	90 (75-100)
After negative test result	0-0.11	5 (0.1-20)	5 (0-10)	5 (0-10)
Hypothetical testing situation				
After positive test result	2	95 (95-95)	95 (80-100)	95 (95-100)
After negative test result	0	2 (0-10)	5 (0-5)	5 (5-75)

Abbreviation: IQR, interquartile range.

For pneumonia, the median clinical scenario–based estimate of pretest probability by participants was 80% (IQR, 75%-90%; evidence range, 25%-42%; *P* < .001). Median estimated probability of pneumonia was 95% (IQR, 90%-100%; evidence range, 46%-65%; *P* < .001) after a positive radiology result and 50% (IQR, 30%-80%; evidence range, 10%-19%; *P* < .001) after a negative radiology result. After a positive radiology result, 551 practitioners (99.6%) would treat with antibiotics, whereas 401 (72.5%) would treat with antibiotics after a negative radiology result.

For breast cancer, the clinical scenario–based estimate of pretest probability by participants was 5% (IQR, 1%-10%; evidence range, 0.2%-0.3%; *P* < .001). Median estimated probability of breast cancer was 50% (IQR, 30%-80%; evidence range, 3%-9%; *P* < .001) after a positive mammography result and 5% (IQR, 1%-10%; evidence range, <0.05%; *P* < .001) after a negative mammography result.

For cardiac ischemia, the median clinical scenario–based estimate of pretest probability by participants was 10% (IQR, 5%-20%; evidence range, 1%-4.4%; *P* < .001). The median estimated probability of cardiac ischemia was 70% (IQR, 50%-90%; evidence range, 2%-11%; *P* < .001) after a positive exercise stress test result and 5% (IQR, 1%-10%; evidence range, 0.43%-2.5%; *P* < .001) after a negative exercise stress test result. After a positive test result, 432 (78.1%) would treat for cardiac ischemia.

For UTI, the description was of asymptomatic bacteriuria. The median clinical scenario–based estimate of pretest probability by participants was 20% (IQR, 10%-50%; evidence range, 0%-1%; *P* < .001). The median estimated probability of a UTI was 80% (IQR, 30%-95%; evidence range, 0%-8.3%; *P* < .001) after a positive urine culture result and 5% (IQR, 0%-10%; evidence range, 0%-0.11%; *P* < .001) after a negative urine culture result. After a positive test result, 393 (71.1%) would treat with antibiotics. After a negative test result, 43 practitioners (7.8%) would treat with antibiotics.

Scenarios requesting identical test interpretation based on hypothetical numbers revealed similar tendencies. For the question, “A test to detect a disease for which prevalence is 1 out of 1000 has a sensitivity of 100% and specificity of 95%. What is the chance that a person found to have a *positive* result actually has the disease?” the median answer was 95% (IQR, 95%-100%), whereas the correct answer was 2%. For the related question, “What is the chance that a person found to have a *negative* result actually has the disease?” the median answer was 5% (IQR, 0%-5%), whereas the correct answer was 0%.

### Imputed Likelihood Ratios

Imputed likelihood ratios were of variable accuracy across clinical scenarios. The most accurate were those for the impact of chest radiography for the diagnosis of pneumonia and urine culture for the diagnosis of UTI; the least accurate were those for negative mammography results for breast cancer and positive exercise stress test results for cardiac ischemia (imputed median positive and negative likelihood ratios for practitioners for chest radiography for pneumonia: positive likelihood ratio, 4.8; evidence, 2.6; negative likelihood ratio, 0.3; evidence, 0.3; those for mammography for breast cancer: positive likelihood ratio, 44.3; evidence range, 13.0-33.0; negative likelihood ratio, 1.0; evidence range, 0.05-0.24; those for exercise stress test for cardiac ischemia: positive likelihood ratio, 21.0; evidence range, 2.0-2.7; negative likelihood ratio, 0.6; evidence range, 0.5-0.6; those for urine culture for urinary tract infection: positive likelihood ratio, 9.0; evidence, 9.0: negative likelihood ratio, 0.1; evidence, 0.1.) (Table 4). Estimates of probability and imputed likelihood ratios were similar between residents and primary care practitioners (Table 4).

**Table 4.** Imputed Positive and Negative Likelihood Ratios Calculated for Each Practitioner Based on Their Pretest and Posttest Positive or Negative Responses

Test and LR result	Scientific evidence of likelihood ratio	Imputed LR, median (IQR)		
		Resident physician	Attending physician	Nurse practitioner or physician assistant
Chest radiography for pneumonia				
Positive	2.57	4.75 (2.25-11.00)	5.21 (2.25-52.58)	9.00 (2.15-111.00)
Negative	0.33	0.35 (0.12-0.67)	0.21 (0.06-0.44)	0.25 (0.06-0.42)
Mammography for breast cancer				
Positive	13.00-33.00	36.00 (9.00-196.00)	54.79 (13.22-428.14)	19.00 (4.00-49.00)
Negative	0.05-0.24	1.00 (0.47-1.26)	1.00 (0.44-1.00)	1.00 (0.33-1.56)
Exercise stress test for cardiac ischemia				
Positive	2.03-2.65	22.67 (8.12-81.00)	19.00 (5.44-73.50)	51.00 (9.00-218.50)
Negative	0.51-0.56	0.50 (0.17-1.00)	0.58 (0.11-1.00)	0.59 (0.26-1.00)
Urine culture for UTI				
Positive	9.00	5.10 (1.89-44.33)	16.86 (3.00-147.00)	27.00 (8.14-333.00)
Negative	0.11	0.16 (0.02-0.58)	0.11 (0.01-0.47)	0.12 (0.01-0.63)

Abbreviations: IQR, interquartile range; LR, likelihood ratio; UTI, urinary tract infection.

## Discussion

In this survey study, in scenarios commonly encountered in primary care practice, practitioners overestimated the probability of disease by 2 to 10 times compared with the scientific evidence, both before and after testing. This result was mostly associated with overestimates of pretest probability, which were observed across all scenarios. Adjustments to probability in response to test results varied from accurate to overestimates of risk by type of test. There was variation in accuracy between type of practitioner that was small compared with the magnitude of difference between practitioners and the scientific evidence. Many practitioners reported that they would treat patients for disease for which likelihood had been overestimated.

The most striking finding from this study was that practitioners consistently and significantly overestimate the likelihood of disease. Small studies with limited generalizability have had similar findings, often asking practitioners to perform one isolated aspect of diagnosis, such as interpreting a test result. However, past studies^8,9,10,11^ have not explored a range of questions or clarified estimates at different steps in the diagnostic pathway. The reason for inaccurate estimates of probability are not clear, although anecdotes reported during the current study imply that practitioners often do not think in terms of probability. One participant stated that estimating probability of disease “isn’t how you do medicine.” This attitude is consistent with a previous study^20^ of diagnostic strategies that describe an initial pattern recognition phase of care with only 10% of practitioners engaging in a secondary phase of probabilistic reasoning.

This study found that probability estimates were consistently biased toward overestimation, as has been seen in other contexts, such as expectations of high stock returns among investors.^21^ This overestimation is consistent with cognitive biases, including base rate neglect, anchoring bias, and confirmation bias.^14^ These biases drive overestimation because true base rates are usually lower than expected and anchoring tends to reflect experiences that represent improbable events or those in which a diagnosis was missed. Such cognitive biases have been associated with diagnostic errors that may occur from errors in estimating risk.^5,22,23^ Notably, practitioners in this survey were often residents or academic physicians who often practice with populations with higher prevalence of disease. This experience may have also contributed to higher estimates of disease.

Pretest probabilities were consistently overestimated for all questions, but overestimates were particularly apparent for the pneumonia and UTI scenarios. Estimates of pretest probability generally reflect clinical knowledge. Reasons for overestimates for these infectious diseases may relate to the fact that antibiotics are often appropriately given even when the likelihood of infection is moderate. In the UTI scenario, estimates of high pretest probability may reflect the evolution of the definition of asymptomatic bacteriuria as a separate entity from UTI.^24^

In contrast to past literature,^8,9,10,19^ practitioners accurately adjusted estimates of disease based on the results of some tests, as demonstrated by the imputed likelihood ratios. This adjustment could be artifactual because of inability to adjust probability for tests that had high pretest estimates (ie, pneumonia and UTI). In other cases, practitioners markedly overestimated the probability of disease after testing, specifically after a positive or negative mammography result or a positive exercise stress test result. Practitioners are known to overestimate chance of disease when completing a theoretical estimate of likelihood of disease after a positive test result when pretest probability was 1 in 1000 tests.^9,10^ The current study included the identical question with an identical response, with participants estimating the likelihood of disease at 95% when the correct answer was 2%.^5,8,9,10,19^ The findings regarding real-life examples are consistent with evidence from limited past studies,^8,9,10,11^ for example, physician interpretation of a positive mammography result in a typical woman as conveying 81% probability of breast cancer.^8^

The assessment of test results in this study was simplified to positive or negative. This dichotomization reflects the literature on the sensitivity and specificity of testing.^5,6^ However, in clinical medicine, these tests often present a range of descriptions for a positive result from mild positives, such as well-circumscribed density on a mammogram, to a strongly positive result, such as inducible ischemia on a stress test or spiculated mass on a mammogram. A more strongly positive or abnormal result would be less sensitive but more specific for disease. This study did not evaluate interpretation of more complex test results.

There are important implications of the finding of a gap between practitioner estimates and scientific estimates of the probability of disease. Practitioners who overestimate the probability of disease would be expected to use that overestimation when deciding whether to initiate therapy, which could lead to overuse of medications and procedures with associated patient harms. Practitioners in the study reported that they would initiate treatment based on estimates of disease, including 78.2% who would treat cardiac ischemia and 71.0% who would treat a UTI when a positive test result would place their patient at 11% or less chance of disease. These errors would similarly corrupt shared decision-making with patients, which relies on practitioner understanding and communication of the likelihood of various outcomes.^25,26,27^ Training in shared decision-making has focused on communication skills,^28^ not on understanding the probability of disease,^29^ but the findings suggest another important educational target.

More focus on diagnostic reasoning in medical education is important. The finding of a primary problem with pretest probability estimates may be more amenable to intervention than the more commonly discussed bayesian adjustment to probability from test results.^30^ Pretest probability is commonly discussed in medical education, but a standard method for estimating pretest probability has not been described.^30^ Ideally, such estimates incorporate knowledge of disease prevalence and the predictive value of components of the history and physical examination, but for many conditions this information is difficult to find. The fact that estimates are so far from scientific evidence identifies a pressing need for improvement. There are a limited number of well-characterized diseases with pretest probability calculators, notably cardiac ischemia.^31,32^ Despite the fact that respondents in this study had no access to external aids while completing the survey, pretest estimates of cardiac ischemia were more accurate than for other clinical scenarios, implying that access to these calculators may improve knowledge and impact clinical reasoning. There is also a need to improve bayesian adjustment in probability from test results, which requires readily accessible references for clinical sensitivity and specificity. Computer visual decision aids that guide estimates of probability may also have a role.^5,33^ Alternative approaches, such as natural frequencies and naturalistic decision-making or use of heuristics, may improve decisions.^34^

### Limitations

This study has limitations. One is that the small fraction of respondents who did not complete the survey were more likely to be female, nurse practitioners, or physician assistants or to have been in practice for more than 10 years. However, the overall response rate was high. The format of survey questions required participants to estimate pretest probability before giving interpretation of positive or negative test results, which may not reflect their natural practice. Finally, although validity was extensively assessed via a multidisciplinary expert panel, reliability of our novel survey was not assessed.

## Conclusions

In this study, large overestimates of the probability of disease before and after diagnostic testing were observed. Probability adjustments in response to test results varied from accurate to overestimates of risk by type of test. This significant overestimation of disease likely limits the ability of practitioners to engage in precise and evidence-based medical practice or shared decision-making.

## Notes

Supplement. **eAppendix 1.** Survey Questions **eAppendix 2.** Best Answers for Testing Questions
