# Supplementary material for: Accuracy of Practitioner Estimates of Probability of Diagnosis Before and After Testing
Source: JAMA Intern Med. 2021 Apr 5;181(6):747–55. doi: 10.1001/jamainternmed.2021.0269 (PMC8022260; doi:10.1001/jamainternmed.2021.0269)
Supplement: Supplement. — eAppendix 1. Survey Questions eAppendix 2. Best Answers for Testing Questions [file jamainternmed-e210269-s001.pdf]

Supplemental Online Content

Morgan DJ, Pineles L, Owczarzak, et al. Accuracy of Practitioner Estimates of Probability of Diagnosis Before and After Testing. Published online April 5, 2021. *JAMA Internal Medicine*. doi:10.1001/jamainternmed.2021.0269

eAppendix 1. Survey Questions
eAppendix 2. Best Answers for Testing Questions

This supplemental material has been provided by the authors to give readers additional information about their work.

© 2021 Morgan DJ et al. *JAMA Internal Medicine*.

Appendix 1. Survey questions

1. **Ms. Smith, a previously healthy 35-year-old woman who smokes tobacco presents with five days of fatigue, productive cough, worsening shortness of breath, fevers to 102ºF and decreased breath sounds in the lower right field. She has a heart rate of 105 but otherwise vital signs are normal. She has no particular preference for testing and wants your advice.**

   How likely is it that Ms. Smith has pneumonia based on this information? _______%

   Ms. Smith's chest **X-ray is consistent with pneumonia**. How likely is she to have pneumonia? _______%

   Ms. Smith's chest **X-ray is negative**. How likely is she to have **pneumonia**? _______%

You are seeing Ms. Johnson, 45-year-old woman, for an annual visit. She has no specific risk factors or symptoms for breast cancer. She has no particular preference for testing and wants your advice.

How likely is Ms. Johnson to have breast cancer based on this information? _______%

Ms. Johnson's **mammogram is positive**. How likely is she to have breast cancer? _______%

Ms. Johnson's **mammogram is negative**. How likely is she to have breast cancer? _______%

2. **You are seeing Mrs. Jones, a 43-year-old premenopausal woman with atypical chest pain and a normal ECG. She has no risk factors and normal vital signs/examination. She has no particular preference for testing and wants your advice.**

   How likely is Mrs. Jones' to have cardiac ischemia based on this information? _______%

   Mrs. Jones' **exercise stress test is positive.** How likely is she to have cardiac ischemia? _______%

   Mrs. Jones' **exercise stress test is negative**. How likely is she to have cardiac ischemia? _______%

3. **Mr. Williams, a 65-year-old man, comes to the office for follow up of his osteoarthritis. He has noted foul-smelling urine and no pain or difficulty with urination. A urine dipstick shows trace blood. He has no particular preference for testing and wants your advice.**

   How likely is Mr. Williams to have a urinary tract infection (UTI)? _______%

   Mr. Williams' **urine culture is positive**. How likely is he to have a UTI? _______%

   Mr. Williams' **urine culture is negative**. How likely is he to have a UTI? _______%

4. **A test to detect a disease for which prevalence is 1 out of 1000 has a sensitivity of 100% and specificity of 95%.**

   What is the chance that a person found to have a **positive** result actually has the disease? _______%

   What is the chance that a person found to have a **negative** result actually has the disease? _______%

© 2021 Morgan DJ et al. *JAMA Internal Medicine*.

Appendix 2. Best answers for testing questions

To identify the best evidence-based pretest probability, sensitivity and specificity from the literature, we used a hierarchical method.

1. Data was first sought from high-quality recent systematic reviews, meta-analyses, and/or guidelines.
2. If only older systematic reviews, meta-analyses, and/or guidelines were available with newer high-impact studies after publication, we considered data from both (attempting to understand most accurate numbers for current technology/practice)
3. If no systematic reviews, meta-analyses, and/or guidelines were available, we used data from commonly cited studies based on citations in recent guidelines and creating weighted averages by consensus. The expert panel of physicians overseeing the study was presented with best evidence identified and settled on evidence-based answers presented in results.

© 2021 Morgan DJ et al. *JAMA Internal Medicine*.

Question 1

Ms. Smith, a previously healthy 35-year-old woman who smokes tobacco presents with five days of fatigue, productive cough, worsening shortness of breath, fevers to 102°F and decreased breath sounds in the lower right field. She has a heart rate of 105 but otherwise vital signs are normal. She has no particular preference for testing and wants your advice.

a. How likely is it that Ms. Smith has pneumonia based on this information? (pretest probability)
35-yo woman, fatigue, cough, SOB, fevers 102°F/38.9°C, tachycardia

There are no systematic reviews/meta-analyses for pretest probability. The closest thing we could identify was Metlay et al.[1]

Prevalence—starting pretest, **5% of all patients visiting primary care physicians for cough diagnosed as having pneumonia**.Heckerling[2] references the National Health Survey; 3% of people with respiratory infections have PNA.

<u>Prediction Rules</u>

All prediction rules are developed compared to their ability to identify infiltrates on CXR.

Pretest probability is best determined by pneumonia prediction algorithms. There are a few:

- **Heckerling criteria**[2]—based on the absence of asthma, temp >100°F, HR >100, decreased BS (this patient was missing one variable of crackles) she has a **25%** chance of pneumonia.
    - Article complicated but has a nomogram. Assuming 5% prevalence of PNA in primary care, 4 RF = **25%** probability of pneumonia.

- **Diehr criteria**[3]—sputum, temp >100 (3 points) Score of +3 = +LR 14—with same 5% prevalence = 42%

We looked for more recent publications and the only one identified was Tse et al.[4] This isn't as well developed and is confusing to interpret. Making some assumptions about duration of fever >3 days, it would predict ~33% pretest probability of pneumonia, so wouldn't change the conclusion from the Heckerling & Diehr criteria.

In summary, pretest probability 25-42%

There are no systematic reviews/meta-analyses for sensitivity & specificity of a chest x-ray for a clinical diagnosis of pneumonia. The best systematic review was Ye et al.[5], which was primarily focused on using lung ultrasound for the diagnosis of CAP. It provides sensitivity & specificity, but this is vs. hospital ICD code, which is likely very non-specific for pneumonia.

The most informative paper for determining sensitivity and specificity was Claessens et al.[6] This was a prospective cohort study performed in the ER.

- 319 patients with suspected CAP prospectively enrolled from ER.
- Classified as definite, probable/possible or excluded clinically for CAP → had immediate CXR then CT. Report rates of changing diagnoses.
- 188 had positive CXR.
- 40 (33%) with negative CXR had infiltrate on CT
    - Of all 188 positive CXR, 131 were true positive, 56 false positive → False positive rate = 56/188 (29.9%) **(Spec 70.1%) (all with CT as gold standard)**
- Of all patients with pneumonia by CT, 132 had positive CXR and 40 had false negative, so **CXR 76.7% sensitive**

A similar article that was reviewed but ultimately not referred to is: Self et al[7] This was a convenience sample of patients evaluated for PE or other non PNA reason who had a CXR and CT scan, we don't think this is as helpful as Claessens and haven't included the data.

© 2021 Morgan DJ et al. *JAMA Internal Medicine*.

We should note that the threshold for action for treatment of suspected pneumonia is often a relatively low pretest probability (or positive predictive value).

CXR for community acquired pneumonia	
**Sensitivity**	**76.7%**
**Specificity**	**70.1%**
**Positive likelihood ratio**	**2.57**
**Negative likelihood ratio**	**0.33**

b. Ms. Smith's chest X-ray is consistent with pneumonia. How likely is she to have pneumonia? (PPV)
46%-65% PPV (calculation based on pretest probability, sensitivity/specificity)

c. Ms. Smith's chest X-ray is negative. How likely is she to have pneumonia? (posterior probability if test negative, 1-NPV)
10%-19%

Question 1 References

1. Metlay JP, Kapoor WN, Fine MJ. Does this patient have community-acquired pneumonia? Diagnosing pneumonia by history and physical examination. JAMA. 1997;278(17):1440-1445.
2. Heckerling PS, Tape TG, Wigton RS, et al. Clinical prediction rule for pulmonary infiltrates. Ann Intern Med. 1990;113(9):664-670. doi:10.7326/0003-4819-113-9-664
3. Diehr P, Wood RW, Bushyhead J, Krueger L, Wolcott B, Tompkins RK. Prediction of pneumonia in outpatients with acute cough--a statistical approach. J Chronic Dis. 1984;37(3):215-225. doi:10.1016/0021-9681(84)90149-8
4. Tse CF, Chan YYF, Poon KM, Lui CT. Clinical prediction rule to predict pneumonia in adult presented with acute febrile respiratory illness. Am J Emerg Med. 2019;37(8):1433-1438. doi:10.1016/j.ajem.2018.10.039
5. Ye X, Xiao H, Chen B, Zhang S. Accuracy of Lung Ultrasonography versus Chest Radiography for the Diagnosis of Adult Community-Acquired Pneumonia: Review of the Literature and Meta-Analysis. PLoS ONE. 2015;10(6):e0130066. doi:10.1371/journal.pone.0130066
6. Claessens Y-E, Debray M-P, Tubach F, et al. Early Chest Computed Tomography Scan to Assist Diagnosis and Guide Treatment Decision for Suspected Community-acquired Pneumonia. Am J Respir Crit Care Med. 2015;192(8):974-982. doi:10.1164/rccm.201501-0017OC
7. Self WH, Courtney DM, McNaughton CD, Wunderink RG, Kline JA. High discordance of chest x-ray and computed tomography for detection of pulmonary opacities in ED patients: implications for diagnosing pneumonia. Am J Emerg Med. 2013;31(2):401-405. doi:10.1016/j.ajem.2012.08.041

© 2021 Morgan DJ et al. *JAMA Internal Medicine*.

Question 2

You are seeing Ms. Johnson, 45-year-old woman, for an annual visit. She has no specific risk factors or symptoms for breast cancer. She has no particular preference for testing and wants your advice.

a. **How likely is Ms. Johnson to have breast cancer based on this information? (Pretest Probability)**
According to the most recent ACS breast cancer statistics[8], the 10-year probability of developing invasive breast cancer is 1.5% for women age 40-50. If we divide by year, we get 1.5/10 = **0.15%** per year, <1% at any one point.

Banks et al. 2004[9]: 122,355 women aged 50-64 (higher risk age) found **0.59% pretest prob** for each mammogram

Barlow et al. 2004[10]: 469,512 women from BCS consortium, **0.3% pretest prob** (3/1000 mammograms have cancer)

In summary, **~0.2-0.3% pretest prob for a mammogram** (>0.15% of ACS, at Barlow rate. Banks is higher risk group)

USPSTF evidence summary[11]
"Sensitivity ranges overall from 77-95% and specificity ranges from 94-97%"

Although others have estimated lower specificity, USPSTF seems like an appropriate source and favor going with USPSTF.

**Mammogram for breast cancer**	
**Sensitivity**	**77-95%**
**Specificity**	**94-97%**
**Positive likelihood ratio**	**13-33**
**Negative likelihood ratio: 0.16**	**.05-0.24**

b. **Ms. Johnson's mammogram is positive. How likely is she to have breast cancer? (PPV)**
PPV of 3-9%

c. **Ms. Johnson's mammogram is negative. How likely is she to have breast cancer?** (posterior probability if test negative, 1-NPV)
Pretty close to zero (<0.05%)

Question 2 References

8. DeSantis CE, Ma J, Gaudet MM, et al. Breast cancer statistics, 2019. CA: A Cancer Journal for Clinicians. 2019;69(6):438-451. doi:10.3322/caac.21583
9. Banks E, Reeves G, Beral V, et al. Influence of personal characteristics of individual women on sensitivity and specificity of mammography in the Million Women Study: cohort study. BMJ. 2004;329(7464):477. doi:10.1136/bmj.329.7464.477
10. Barlow WE, Chi C, Carney PA, et al. Accuracy of screening mammography interpretation by characteristics of radiologists. J Natl Cancer Inst. 2004;96(24):1840-1850. doi:10.1093/jnci/djh333
11. Siu AL, U.S. Preventive Services Task Force. Screening for Breast Cancer: U.S. Preventive Services Task Force Recommendation Statement. Ann Intern Med. 2016;164(4):279-296. doi:10.7326/M15-2886

Question 3

You are seeing Mrs. Jones, a 43-year-old premenopausal woman with atypical chest pain and a normal ECG. She has no risk factors and normal vital signs/examination. She has no particular preference for testing and wants your advice.

a. **How likely is Mrs. Jones' to have cardiac ischemia based on this information? (Pretest Probability)**

© 2021 Morgan DJ et al. *JAMA Internal Medicine*.

We identified a systematic review in JAMA Rational Clinical Examination series by Fanaroff et al in 2015.[12] This review discussed clinical prediction rules. This was focused on the ER where the overall prevalence of acute coronary syndrome was estimated to be 13% (relatively high, and not the setting for this patient). However, we believe this is worth discussing. This patient would be categorized as LOW risk by all tools discussed, although some rules require obtaining a troponin test to categorize patients. The risk of a patient like this, considering 13% prevalence of ACS in ER would be 2.9% to 4.4%. Using prevalence of acute coronary syndrome in primary care with these prediction rules would lead to estimates of 1-2.7% pretest probability.[13]

For a general discussion of calculators, see DiCarli et al.[14] Some cohorts are discussed for pretest probability by UpToDate but they only include those who had angiography and therefore have higher average pretest probability.

The best performing model seems to be the CAD consortium score[15]. This is the European CAD consortium score that was shown to be better than Diamond & Forrester.[16,17] For this model, apparently the best, the basic model gives a 3% risk whereas the more nuanced clinical model provides a 1% pretest probability.

<u>Other scores reviewed</u>:
PreTest Consult score[18] was used by Victor Montori in a randomized trial;[19] it is simple and attributes 0.6% risk for this patient.

Diamond-Forrester initial model (1979)[20]
https://fpnotebook.com/CV/Exam/DmndAndFrstrChstPnPrdctnRl.htm Duke Clinical Score
https://zunis.org/Duke%20Chest%20Pain%20-%20CAD%20Predictor.htm 2-3% pretest probability

In summary, pretest probability for this patient is low.
1% from CAD consortium clinical model is best, but would allow up to 4.4% given Duke, HEART, TIMI and other commonly discussed scores.
Pretest 1-4.4%

The most recent guideline identified was Fihn et al.[21] This has clear summaries of the sensitivity and specificity of multiple cardiology tests. For the exercise ECG test they state: "The composite diagnostic sensitivity and specificity, unadjusted for referral bias, is 61% and ranges from 70% to 77%, but it is lower in women."

**Exercise stress ECG test**	
**Sensitivity**	**61%**
**Specificity**	**70-77%**
**Positive likelihood ratio**	**2.03-2.65**
**Negative likelihood ratio**	**0.51-0.56**

b. Mrs. Jones' exercise stress test is positive. How likely is she to have cardiac ischemia? (PPV)
PPV: 2% to 11%

c. Mrs. Jones' exercise stress test is negative. How likely is she to have cardiac ischemia? (posterior probability if test negative, 1-NPV)
Posterior probability, if negative: 0.43%-2.5%

Question 3 References

12. Fanaroff AC, Rymer JA, Goldstein SA, Simel DL, Newby LK. Does This Patient With Chest Pain Have Acute Coronary Syndrome?: The Rational Clinical Examination Systematic Review. *JAMA*. 2015;314(18):1955-1965. doi:10.1001/jama.2015.12735
13. Yazdani S, Hosseinzadeh M, Hosseini F. Models of clinical reasoning with a focus on general practice: A critical review. *J Adv Med Educ Prof*. 2017;5(4):177-184.

© 2021 Morgan DJ et al. *JAMA Internal Medicine*.

14. Di Carli MF, Gupta A. Estimating Pre-Test Probability of Coronary Artery Disease: Battle of the Scores in an Evolving CAD Landscape. *JACC Cardiovasc Imaging*. 2019;12(7 Pt 2):1401-1404. doi:10.1016/j.jcmg.2018.04.036
15. Pre-test probability of CAD (CAD consortium). Calculate by QxMD. Accessed January 8, 2020. https://qxmd.com/calculate
16. Genders TSS, Steyerberg EW, Alkadhi H, et al. A clinical prediction rule for the diagnosis of coronary artery disease: validation, updating, and extension. *Eur Heart J*. 2011;32(11):1316-1330. doi:10.1093/eurheartj/ehr014
17. Genders TSS, Steyerberg EW, Hunink MGM, et al. Prediction model to estimate presence of coronary artery disease: retrospective pooled analysis of existing cohorts. *BMJ*. 2012;344:e3485. doi:10.1136/bmj.e3485
18. PREtestConsultACS. Accessed January 8, 2020. http://s6.studymaker.com/pretest/quadricp2/acs/acs.php?PTPAuth=QUADRIC
19. Hess EP, Knoedler MA, Shah ND, et al. The chest pain choice decision aid: a randomized trial. *Circ Cardiovasc Qual Outcomes*. 2012;5(3):251-259. doi:10.1161/CIRCOUTCOMES.111.964791
20. Diamond and Forrester Chest Pain Prediction Rule. Accessed January 8, 2020. https://fpnotebook.com/CV/Exam/DmndAndFrstrChstPnPrdctnRl.htm
21. Fihn SD, Gardin JM, Abrams J, et al. 2012 ACCF/AHA/ACP/AATS/PCNA/SCAI/STS Guideline for the diagnosis and management of patients with stable ischemic heart disease: a report of the American College of Cardiology Foundation/American Heart Association Task Force on Practice Guidelines, and the American College of Physicians, American Association for Thoracic Surgery, Preventive Cardiovascular Nurses Association, Society for Cardiovascular Angiography and Interventions, and Society of Thoracic Surgeons. *J Am Coll Cardiol*. 2012;60(24):e44-e164. doi:10.1016/j.jacc.2012.07.013

© 2021 Morgan DJ et al. *JAMA Internal Medicine*.

Question 4

Mr. Williams, a 65-year-old man, comes to the office for follow up of his osteoarthritis. He has noted foul-smelling urine and no pain or difficulty with urination. A urine dipstick shows trace blood. He has no particular preference for testing and wants your advice.

a. **How likely is Mr. Williams to have a urinary tract infection (UTI)? (Pretest Probability)**
Mr. Williams has symptoms most compatible with asymptomatic bacteriuria, based on lack of any definite symptoms of UTI. This interpretation is consistent with Infectious Disease Society of America (IDSA) guideline for UTI[22] as well as IDSA Asymptomatic Bacteriuria (ASB) guideline.[23] This was interpreted to mean his chance of UTI included 0%. However, given small risk of asymptomatic bacteriuria to develop into pyelonephritis in specific groups[24] and clinical experience of rare cases of complicated UTI with bacteremia without symptoms localizing to the urinary tract, probability was expanded to be a range form 0-1%.

Pretest probability = 0-1%

The sensitivity and specificity of urine culture for diagnosis of urinary tract infection (UTI) in different patient populations was obtained from a systematic review to augment current IDSA guidelines.[25] A partial table from that review is below.

Patient population	Sensitivity	Specificity
Healthy outpatient women[26–30]	90%	86%

**Urine culture for UTI**	
**Sensitivity**	**90%**
**Specificity**	**90%**
**Positive likelihood ratio**	**9**
**Negative likelihood ratio**	**0.11**

b. **Mr. Williams' urine culture is positive. How likely is he to have a UTI? (PPV)**
Correct answer is 0-8.3%

c. **Mr. Williams' urine culture is negative. How likely is he to have a UTI?** (posterior probability if test negative, 1-NPV)
0-0.11%

Question 4 References

22. Hooton TM, Gupta K. Acute complicated urinary tract infection (including pyelonephritis) in adults. UpToDate. Accessed January 9, 2020. https://www.uptodate.com/contents/acute-complicated-urinary-tract-infection-including-pyelonephritis-in-adults?search=pyuria%20adult&source=search_result&selectedTitle=6~150&usage_type=default&display_rank=6
23. Nicolle LE, Gupta K, Bradley SF, et al. Clinical Practice Guideline for the Management of Asymptomatic Bacteriuria: 2019 Update by the Infectious Diseases Society of America. *Clin Infect Dis*. 2019;68(10):1611-1615. doi:10.1093/cid/ciz021
24. Henderson JT, Webber EM, Bean SI. Screening for Asymptomatic Bacteriuria in Adults: Updated Evidence Report and Systematic Review for the US Preventive Services Task Force. *JAMA*. 2019;322(12):1195-1205. doi:10.1001/jama.2019.10060
25. Chan-Tack KM, Trautner BW, Morgan DJ. The varying specificity of urine cultures in different populations. *Infect Control Hosp Epidemiol*. Published online February 10, 2020:1-2. doi:10.1017/ice.2020.16
26. Córdoba G, Holm A, Sørensen TM, et al. Use of diagnostic tests and the appropriateness of the treatment decision in patients with suspected urinary tract infection in primary care in Denmark - observational study. *BMC Fam Pract*. 2018;19(1):65. doi:10.1186/s12875-018-0754-1

© 2021 Morgan DJ et al. *JAMA Internal Medicine*.

27. Pernille H, Lars B, Marjukka M, Volkert S, Anne H. Sampling of urine for diagnosing urinary tract infection in general practice - First-void or mid-stream urine? *Scand J Prim Health Care*. 2019;37(1):113-119. doi:10.1080/02813432.2019.1568708
28. Hooton TM, Roberts PL, Cox ME, Stapleton AE. Voided midstream urine culture and acute cystitis in premenopausal women. *N Engl J Med*. 2013;369(20):1883-1891. doi:10.1056/NEJMoa1302186
29. Walter FG, Knopp RK. Urine sampling in ambulatory women: midstream clean-catch versus catheterization. *Ann Emerg Med.* 1989;18(2):166-172. doi:10.1016/s0196-0644(89)80108-8
30. Stamm WE, Counts GW, Running KR, Fihn S, Turck M, Holmes KK. Diagnosis of coliform infection in acutely dysuric women. *N Engl J Med*. 1982;307(8):463-468. doi:10.1056/NEJM198208193070802

© 2021 Morgan DJ et al. *JAMA Internal Medicine*.
